# Inverse care and the role of the state: the health of the urban poor

**DOI:** 10.2471/BLT.16.179325

**Published:** 2017-02-01

**Authors:** Devaki Nambiar, Harsh Mander

**Affiliations:** a Public Health Foundation of India, Plot No. 47, Sector 44, Institutional Area Gurgaon, 122002 Delhi NCR, India.; b Centre For Equity Studies, Delhi, India.

Tudor Hart’s inverse care law, set out in an article in *The Lancet* in 1971, states that the availability of good medical care tends to vary inversely with the need of the population served.^1^ Referring to the national health service (NHS) of the United Kingdom of Great Britain and Northern Ireland, the author argued that the state must play a role in ensuring the health and well-being of those excluded by a range of larger social and market forces. Despite concerns that its services are being eroded, the NHS still adheres to the principle of state provision of publicly funded health care for all.

Worldwide, our cities and towns are a growing example of the inverse care law. The inequalities in health-seeking and health outcomes among city sub-populations are well documented.^2^ In our decade-long experience working in urban health care in India we found that all too often the state has played a role in furthering the exclusion of the urban poor, with deleterious impacts on their health and health-seeking. Here, we describe this tendency, its impact and what can be done about it.

In many parts of the world, cities open up opportunities for making a living, however basic, for people from rural areas. Cities can also offer an escape from the constraints of patriarchy-, gender-, caste- and race-based hierarchies, and this can have positive ramifications for health.^3^ However, in many low- and middle-income countries, the transition from a rural to urban existence is part of a larger process of neoliberal economic reform.^4^ The result is that the state neglects rural development, thus driving migration to urban areas, but also underfunds urban social welfare, creating pockets of deprivation alongside concentrations of private wealth in towns and cities.^2^^,^^3^^,^^5^

What aggravates the predicament of poor migrants to cities is that they are often regarded as illegitimate non-citizens by the state, even though their contributions to building and running our cities are indispensable.^4^ In countries like China and Viet Nam, systems of household registration used to present legal barriers to the entry of many poor persons into cities. By contrast, in India the constitution of the country guarantees the right of any citizen to move to, live and work in any part of the country. Yet various policies suggest a hostile attitude of state authorities towards migrants.^6^ Across many low- and middle-income countries, the state does not fulfil a positive duty towards rural-to-urban migrants, such as extending health care, education or social security services to them; rather it acts in a negative way by removing rights, banning livelihoods and demolishing homes.

A state that is actively hostile to its most vulnerable urban residents uses many strategies to perpetuate exclusion. One of these is simply making certain urban populations invisible. Across cities in Africa, Asia and Latin America, street children and homeless adults have for generations lived precarious, sometimes violent, and unprotected lives. In a literal sense, they are the most visible of all urban populations, as they have no walls or roof to hide their every move from observers. Yet people of privilege as well as the state treat them as though they do not exist, ignoring any obligations to pursue positive policies for their housing, protection, food and nutrition, health care and education.^4^

Instead, these groups must resort to precarious and exploitative relationships with private intermediaries for shelter, employment and other basic needs.^6^ Other groups who may be made invisible include people living with stigmatized and debilitating ailments, such as leprosy, mental illness, tuberculosis and acquired immunodeficiency syndrome; old people without care; people living in hunger; and children and women facing abuse inside the home. The anonymity of urban life facilitates the invisibility of vulnerable people, which then absolves the state of obligations towards them.

A second, often overlapping, strand of public policy – not just in the industrializing but also the industrialized world – is to treat as illegal the self-help efforts of the urban poor in cities. This includes their efforts towards housing (on the streets, in shanties and informal housing, or Roma caravans) or employment (such as street-vending or the work of undocumented migrants). This adds a layer of social stigma and prejudice to the existing precariousness of these people, and reinforces state neglect, as has been seen with Roma populations across Europe.^7^

This process of illegalization sometimes becomes more vicious when the efforts of the poor to survive and cope are treated as criminal acts. In cities across the globe, working-class areas with high concentrations of ethnic minorities are treated as unsafe and potentially criminal by the state as well as by better-off citizens.^2^^,^^4^ In many countries begging is treated as a crime rather than a pretext for social protection. In Brazil, there were reports of street children being cleared violently from the streets in preparation for the 2016 Olympic Games,^8^ while in Japan, the United States of America, and recently in India, laws have been amended to treat children in conflict with the law as adults. Illegalization and criminalization of these vulnerable groups also results in their being placed in custodial care institutions and residential homes. In these instances, as the cost of state support, the vulnerable person must accept the denial of their freedom, dignity and agency.

In this scenario, impoverished migrants to cities must cope with life under conditions that are precarious by the very design of public policy and law. They are not legally entitled to the housing, sanitation, employment and security that would enable them to have better health.^5^ Even among ethnic, religious or racial minorities that have lived in cities for many years, the passage of time does not automatically mean better provision for younger generations. As an example, in 2010 in the American state of North Carolina, every 10% increase in the African American population census block (representing a population of about 89 600 people) was associated with a 3.8% increase in the odds of exclusion from municipal water services.^9^ In developing countries such as India, open defecation in urban areas occurs among the poorest households, with disproportionate health impacts on child health.^3^ The urban poor also incur greater health expenditure as a proportion of their total expenses when compared with other households.^5^ The inability to access public health facilities for reasons of cost or stigma places the already vulnerable – infants and children, women, the disabled, the chronically ill and elderly people – at a much greater risk or morbidity and mortality.^3^

Exclusion in urban areas is associated with unique and pernicious risks. From the Philippines to South Africa, Brazil to India, slum-dwellers face a greater risk of violence, particularly gender-based, than the general population.^2^ Violence and unrest place an additional toll on mental health and have been associated with impaired cognitive function among the urban poor.^10^ Unsurprisingly, those with stress-filled lives resort to risky behaviour, such as substance abuse and sex work, as a way of coping. According to data published in 2015, the prevalence of smoking among the poorest one fifth of urban men compared with the richest one fifth was more than five times higher in Cambodia and Sierra Leone, with even higher odds in Bangladesh, Indonesia and Malawi.^2^

Increasingly, however, examples are emerging of the state acting to reverse exclusion, by becoming the guarantor and provider of land rights, housing, water, livelihoods, education and health services.^11^ But often it is citizens themselves – the vulnerable and those acting in solidarity with them – who have campaigned for inclusion or played centre-stage in reform efforts. For example, the cities of Mumbai in India and Vinh in Viet Nam have seen some success with slum redevelopment involving federations of slum-dwellers working with municipal authorities.^6^ In Nairobi, public provision of access to water has helped improve sanitation and water access, resulting in reduced under-five mortality and deaths from diarrhoea.^2^ In Cape Town (South Africa), Chicago (USA) and Monrovia (Liberia), pilot projects are underway to provide life-skills training and job opportunities for young urban-dwellers to break cycles of violence.^2^ Public provision of health services through community and neighbourhood clinics is underway in Delhi (India), Guangzhou (China) and Mysore (India), setting a precedent for the state to make visible and to serve urban poor populations, with the participation of citizens.^2^^,^^12^

These examples are rare: the norm remains one in which poor populations in cities continue to be treated as interlopers by the state, always facing the risk of removal and relocation. Tudor Hart argued that “this inverse care law operates more completely where medical care is most exposed to market forces, and less so where such exposure is reduced.”^1^ Nations and global institutions are beginning to openly acknowledge the limitations of market-driven health care and the state’s complicity in the growth of inequality in health outcomes.^13^ We must now, belatedly, embrace the moral imperative for public provision of health care for excluded groups: care that is sensitively designed and delivered with dignity.
